# Barriers and facilitators to effective pain management in elderly Arab patients: a nursing perspective through a qualitative study

**DOI:** 10.1186/s12912-024-02523-6

**Published:** 2024-12-06

**Authors:** Mostafa Shaban, Marwa Mamdouh Shaban, Huda Hamdy Mohammed, Hend Reda Ali El-kest

**Affiliations:** 1 Geriatric Nursing, Faculty of Nursing, Cairo University, Cairo, Egypt; 2 Community Health Nursing- Faculty of Nursing- Cairo University, Cairo, Egypt; 3 Faculty of Nursing, Cairo University, Cairo, Egypt; 4 Community Health Nursing, Faculty of Nursing, Tanta University, Tanta, Egypt

**Keywords:** Pain management, Elderly care, Arab healthcare, Cultural competence, Nursing perspective, Qualitative research

## Abstract

**Background:**

Effective pain management in elderly patients is crucial for quality of life, yet cultural and institutional factors can significantly impact care delivery, particularly in Arab healthcare settings.

**Aim:**

To explore the barriers and facilitators to effective pain management in elderly Arab patients from the perspective of nurses.

**Methods:**

A qualitative descriptive study was conducted with 12 registered nurses from various departments at Tanta University Hospitals, Egypt. Data were collected through semi-structured interviews, observations, and document analysis. Content analysis was used to identify themes and subthemes.

**Results:**

Five main themes emerged: (1) Cultural Barriers to Pain Expression, including stoicism and religious beliefs; (2) Institutional Barriers to Pain Management, such as resource limitations and time constraints; (3) Facilitators to Effective Pain Management, including family support and nurse adaptability; (4) Interdisciplinary Collaboration, emphasizing teamwork and education; and (5) Emotional and Professional Rewards for nurses. Cultural factors often led to underreporting of pain, while institutional constraints hindered thorough assessments. Nurse adaptability and family support, when present, facilitated better pain management.

**Conclusion:**

The study reveals complex interplay between cultural, institutional, and professional factors influencing pain management in elderly Arab patients. Findings suggest the need for culturally sensitive pain assessment tools, enhanced nurse education in pain management, and policies promoting family-centered care and interdisciplinary collaboration.

**Implications:**

Results can inform the development of culturally appropriate pain management strategies and policies in Arab healthcare settings, potentially improving care quality for elderly patients.

**Supplementary Information:**

The online version contains supplementary material available at 10.1186/s12912-024-02523-6.

## Introduction

Pain management in elderly patients is a critical aspect of healthcare, given the high prevalence of chronic pain conditions among the aging population. Effective pain management is particularly challenging in elderly patients due to several factors, including comorbidities, cognitive decline, polypharmacy, and altered pain perception [1]. As global healthcare systems strive to improve the quality of life for elderly individuals, understanding the nuances of pain management is essential [2]. While there is considerable research on pain management in elderly populations, much of the literature has focused on Western contexts, often overlooking the unique sociocultural and healthcare challenges present in the Arab world [3]. Moreover, the role of nurses—often the primary caregivers in managing pain—remains underexplored, particularly in Arab healthcare settings [4].

In the Arab world, healthcare is shaped by cultural values, religious beliefs, and social structures that differ significantly from those in Western societies [5]. The family plays a central role in caregiving, often acting as the intermediary between the patient and healthcare providers [6]. Additionally, cultural attitudes towards pain, suffering, and expressions of discomfort are heavily influenced by religious and societal norms. For example, in many Arab societies, enduring pain with stoicism is viewed as virtuous, and pain may be considered a test of faith [7, 8]. These beliefs can lead to underreporting of pain, reluctance to seek medical help, and a preference for traditional remedies over biomedical interventions. This cultural framework presents unique challenges to healthcare providers, particularly nurses, who are responsible for assessing and managing pain in a clinical setting [9].

Nursing practices in the Arab world are also shaped by systemic and institutional factors. Nurses in this region often face a heavy workload due to understaffing, high patient-to-nurse ratios, and limited resources [10]. These challenges can hinder the ability of nurses to provide comprehensive pain assessments and individualized care. Furthermore, the training and education of nurses in pain management may be inadequate, particularly in relation to the cultural aspects of care [11]. Despite the central role that nurses play in managing pain, there is a lack of research that explores their experiences, perceptions, and challenges in this context, particularly in relation to elderly patients [12, 13].

The existing literature on pain management in elderly patients has largely focused on the physiological and pharmacological aspects of care. Studies have explored the efficacy of different pain relief medications, the impact of comorbidities on pain management, and the challenges of managing pain in patients with cognitive impairments, such as dementia [14–17]. However, the sociocultural dimensions of pain management, particularly in non-Western contexts, remain underexplored. In the Arab world, cultural and religious beliefs play a significant role in shaping patients’ attitudes towards pain and pain relief [18]. For instance, some patients may perceive pain as an inevitable part of aging or as a divine trial, leading them to refuse or delay treatment [19]. These beliefs can complicate the task of healthcare providers, who must navigate cultural sensitivities while ensuring that patients receive adequate pain relief [20].

Another critical gap in the literature pertains to the role of nurses in pain management. Nurses are often the first point of contact for patients experiencing pain, and they play a crucial role in assessing pain, administering medications, and providing non-pharmacological interventions [11, 21]. However, studies that focus on nurses’ experiences and challenges in managing pain, particularly in the Arab context, are limited. Research in Western countries has highlighted several barriers to effective pain management from a nursing perspective, including inadequate training, lack of time, and institutional constraints [22]. While these issues are likely to be present in the Arab world as well, there are additional challenges related to cultural and religious factors that have not been thoroughly investigated [23]. Nurses may encounter difficulties in assessing pain in patients who are reluctant to express their discomfort due to cultural norms. Moreover, language barriers and communication challenges may further complicate pain assessment, particularly in countries where expatriate nurses provide a significant portion of healthcare services [24].

In addition to cultural and communication barriers, institutional factors also play a significant role in shaping pain management practices in Arab healthcare settings [25]. Many hospitals and clinics in the region are under-resourced, and nurses often face high patient loads, making it difficult to provide individualized care [26]. In some cases, there may be a lack of standardized protocols for pain management, leading to inconsistencies in care. Furthermore, the hierarchical nature of healthcare systems in the Arab world, where physicians are often seen as the ultimate authority, may limit the autonomy of nurses in making decisions about pain management [27].

Despite these challenges, there are also several facilitators to effective pain management that could be leveraged to improve care. Family involvement, which is a hallmark of healthcare in the Arab world, can be both a barrier and a facilitator [28]. While family members may sometimes impede pain management by insisting on traditional remedies or discouraging the use of pain medications, they can also play a positive role by providing emotional support, helping to communicate the patient’s needs, and advocating for better care [29]. Additionally, the strong sense of community and social support in Arab societies can help create a more holistic approach to pain management, where the patient’s emotional, spiritual, and physical needs are addressed [30].

Cultural perceptions of pain as a divine test or an aspect of resilience may lead to underreporting and reluctance in seeking pain relief, contrasting with Western practices that encourage open communication of discomfort [7]. Additionally, institutional structures in Arab healthcare settings, including resource limitations and physician-dominated hierarchies, may hinder nurses’ ability to administer optimal pain management, underscoring a distinct context requiring culturally sensitive approaches [31].

While extensive research addresses pain management in elderly patients, studies in Western contexts rarely consider the sociocultural factors and healthcare practices unique to Arab societies. This study fills an important gap by exploring how cultural values and institutional constraints in Arab settings uniquely influence nursing practices in managing pain among elderly patients.

## Aim of the study

The aim of this study is to explore the barriers and facilitators to effective pain management in elderly Arab patients from the perspective of nurses. By investigating the specific challenges and supportive factors encountered by nurses, this study seeks to provide insights into how cultural, institutional, and healthcare practices influence pain management. The findings will contribute to improving nursing strategies, enhancing the quality of care, and informing policies that can address the unique needs of elderly Arab patients.

### Research question

- What are the barriers and facilitators that nurses encounter in managing pain among elderly Arab patients, and how do cultural, institutional, and healthcare practices influence these challenges and opportunities for effective pain management?

### Study design

This study employed a qualitative descriptive design, aligned with the principles of naturalism and constructivism, to explore the barriers and facilitators to effective pain management among elderly Arab patients from the nursing perspective. This design was chosen to gain a deep understanding of the nurses’ lived experiences and the contextual challenges they face in providing care to elderly patients in pain. The study followed the Standards for Reporting Qualitative Research (SRQR) guidelines to ensure methodological rigor and transparency throughout the research process.

### Study setting

The research was conducted in Tanta City, Egypt, within the healthcare facilities affiliated with Tanta University Hospitals, which serve a diverse population, including a significant proportion of elderly patients. These settings were chosen due to their high patient volume, particularly among the elderly, and their provision of a wide range of medical services, including chronic pain management, geriatric care, and palliative care. The hospital environment provided a rich backdrop for examining the challenges faced by nurses in managing pain in elderly patients, particularly in an Arab cultural context, where pain expression and management may differ from Western norms.

### Participants

Participants were recruited using a purposive sampling strategy, selecting registered nurses with at least five years of experience in elderly care. Recruitment focused on nurses from departments closely involved with pain management, including geriatrics, palliative care, and chronic disease units. To promote diversity, we included nurses of different ages, gender, and educational backgrounds. Initial outreach was conducted through departmental meetings and targeted communications approved by hospital administration. The selection criteria ensured that each nurse had a minimum of five years of experience in clinical nursing, with at least three years dedicated to working with elderly patients in healthcare settings such as general wards, outpatient clinics, or palliative care units. This criterion was established to recruit participants with substantial interaction and hands-on experience in managing pain for elderly patients, ensuring that the nurses could provide in-depth insights into both the barriers and facilitators encountered in their daily practice.

Specialties included general medicine, geriatrics, post-operative care, and chronic condition management. This range of specialties allowed the study to capture diverse experiences and strategies related to the management of pain in elderly patients.

In terms of educational background, participants held various qualifications ranging from nursing diplomas to advanced degrees, such as bachelor’s and master’s degrees in nursing. This range in educational attainment contributed to a variety of perspectives on pain management, highlighting differences in clinical approaches and knowledge based on the nurses’ training and academic backgrounds. The study focused exclusively on nurses with direct patient care responsibilities, ensuring that the data reflected real-world interactions and practices related to pain management in elderly patients. Nurses in administrative roles or those without regular contact with patients were excluded to maintain the study’s focus on the clinical aspects of pain management.

### Data collection

Data collection for this study was conducted through face-to-face, in-depth interviews, guided by a semi-structured interview guide developed specifically for the research objectives ( Supplementary Table 1). Each interview session was audio-recorded to capture the entirety of the participants’ responses, with comprehensive notes taken concurrently to document non-verbal cues, reflections, and contextual factors that may have influenced the participants’ statements.

The semi-structured interview guide was carefully designed by the primary author and reviewed by a panel of experts in nursing and communication to ensure that the questions were relevant, comprehensive, and aligned with the study’s aims. The guide focused on topics such as the challenges nurses face in managing pain in elderly patients, the influence of cultural factors on pain management, and the institutional supports or barriers that impact their ability to deliver effective care. Interviews were conducted in a private room within Tanta University Hospitals, ensuring a quiet and comfortable environment where participants could speak freely and without interruptions. Each interview lasted approximately 45 to 60 min.

To accommodate the linguistic preferences of the participants, interviews were conducted in Arabic, as all participants were native speakers. This approach ensured that nurses could express their experiences fully and without the constraints of language barriers. The audio recordings were transcribed verbatim in Arabic by a bilingual nursing researcher who was familiar with healthcare terminology, ensuring that the transcripts accurately reflected the original interviews.

Following transcription, a two-step translation process was implemented to maintain the accuracy and integrity of the data. First, the Arabic transcripts were translated into English by a professional translator with expertise in medical and healthcare contexts. The translated texts were then reviewed by an independent bilingual expert, proficient in both Arabic and English, who compared the translated content against the original transcripts to ensure fidelity in meaning and cultural nuances. Any discrepancies in translation were resolved through discussion, with occasional consultation with the original interviewees to clarify specific statements.

In addition to the interviews, field notes were taken throughout the data collection process. These notes captured non-verbal behaviors, immediate reflections, and environmental observations that were not recorded in the audio. These supplementary notes were reviewed after each interview and incorporated into the data analysis to ensure that important nuances and insights were not overlooked.

The study also employed document reviews and observational data to provide a broader context for understanding the institutional and cultural environment in which the nurses worked. Document reviews included the examination of clinic policies, pain management protocols, and care plans, with permission obtained from the healthcare facilities. This document review helped to identify the formal expectations and guidelines surrounding pain management in elderly patients, providing a framework against which the nurses’ experiences could be compared.

Furthermore, observations were conducted within the hospital settings to witness firsthand the interactions between nurses and elderly patients. These observations focused on the practical application of pain management strategies, the communication between nurses and patients, and the environmental factors influencing care delivery. Observational data were systematically recorded and integrated with the interview and document review findings, allowing for triangulation of data. This comprehensive approach to data collection enhanced the validity and depth of the study’s findings, providing a well-rounded understanding of the barriers and facilitators to effective pain management in elderly Arab patients.

Data management was conducted using a systematic coding process. Transcriptions were anonymized and securely stored in a data management software to maintain confidentiality. To ensure reliability and minimize potential researcher bias, peer debriefing sessions were held regularly, and member checking was employed, allowing participants to review and validate interpretations of their responses.

### Credibility of the study

Ensuring the credibility of this study required a comprehensive approach to data collection and analysis, employing various strategies to validate the findings and enhance the rigor of the research process. This study, which explores the barriers and facilitators to effective pain management in elderly Arab patients from a nursing perspective, established credibility through the following measures:

1. **Prolonged Engagement and Triangulation** Prolonged engagement was achieved by spending extensive time with participants during semi-structured interviews. This interaction allowed the researchers to build rapport with participants and gain a deep understanding of the complexities of pain management in elderly patients, as perceived by nurses. This immersion enriched the data with diverse insights into the cultural, institutional, and clinical challenges faced by nurses in the Arab context. To further strengthen the credibility of the findings, triangulation was employed by cross-referencing data from multiple sources, including interviews, field notes, and document analysis. This triangulation ensured a comprehensive analysis by validating the themes that emerged through multiple perspectives and data sources, each contributing a unique lens to understanding the phenomenon.

2. **Peer debriefing and Member checking** Peer debriefing was incorporated into the study to ensure the accuracy and objectivity of the data analysis. The research team held regular debriefing sessions, during which the initial codes and themes were discussed and refined. This process provided an external check on the data analysis, allowing for the identification of potential biases or blind spots in interpretation. Additionally, member checking was used to further validate the findings. Participants were invited to review summaries of the key themes and interpretations to ensure that these accurately reflected their experiences and perspectives. This feedback process not only confirmed the resonance of the findings with the participants but also helped refine the final themes, grounding the results in the realities of the nurses’ lived experiences.

3. **Thick description** The study utilized thick description to provide a detailed account of the research setting, participant context, and the nuanced interactions observed during data collection. This rich, in-depth portrayal of the cultural and institutional environment in which pain management practices occur allows readers to understand the context-specific factors influencing the findings. Thick description aids in transferring the study’s insights to similar contexts, enhancing the study’s applicability beyond the immediate research setting.

4. **Audit trail** An audit trail was maintained throughout the research process, documenting every step of data collection, analysis, and decision-making. This included records of interview guides, field notes, coding frameworks, and thematic analysis iterations. The audit trail provided transparency, enabling an external reviewer to follow the study’s processes and verify the logic behind coding decisions and theme development. This transparency enhances the trustworthiness of the research findings.

5. **Reflexivity** Reflexivity was practiced throughout the study, with researchers continuously reflecting on their roles, potential biases, and the influence these might have on data interpretation. Reflexive journaling helped researchers remain aware of their preconceptions and cultural perspectives, ensuring that these did not unduly shape the findings. This reflexive approach contributed to a more balanced and objective analysis of the data.

### Data analysis

The data collected from the in-depth, semi-structured interviews were analyzed manually, following the structured content analysis approach outlined by Hsieh and Shannon. This method was selected for its suitability in identifying explicit content while interpreting the latent meanings within the text. The process was carefully designed to ensure a thorough and systematic examination of the interview transcripts, providing a deep understanding of the barriers and facilitators to pain management in elderly Arab patients from the nursing perspective.

### Organizing derived codes

The initial step in the analysis involved familiarization with the interview data. The primary author read the transcripts multiple times to immerse themselves in the content, allowing for the identification of significant segments that directly related to the study’s objectives. Initial codes were derived directly from the text by highlighting key phrases, sentences, or paragraphs that reflected nurses’ experiences, challenges, and strategies in managing pain in elderly patients. These codes captured the essence of each relevant statement and served as the foundation for further analysis.

### Making notes and labeling for codes

As the initial codes were organized, the primary author made extensive notes, reflecting on the context of the statements and considering potential relationships between the codes. This process facilitated deeper analysis beyond surface-level descriptions, ensuring a nuanced interpretation of the data. Codes were systematically labeled to ensure clarity and consistency in categorizing the various aspects of pain management, such as communication challenges, cultural considerations, and institutional barriers. The notes also allowed for tracking emerging ideas that could inform the development of broader themes.

### Emergent categories and cluster formation

Once the codes were organized, they were grouped into emergent categories based on similarities and relationships between them. This step involved an iterative process of comparing and contrasting the codes, refining the categories to ensure that they accurately reflected the data. These categories were then clustered into broader themes that encapsulated the underlying patterns in the participants’ experiences. For instance, codes related to nurses’ difficulty in assessing pain due to cultural beliefs were clustered together to form the theme “Cultural Barriers to Pain Expression.” Similarly, codes addressing the support nurses received from family members were grouped into the theme “Family Involvement as a Facilitator.”

In this study, the relevance of data was determined by its direct connection to the overarching themes related to pain management in elderly Arab patients. For example, a nurse’s reflection on how elderly patients often hesitate to express pain due to cultural beliefs was considered highly relevant. This behavior reflected a significant barrier in assessing pain, which is critical to managing it effectively. Initial codes such as “hesitance to report pain” and “cultural expectation of endurance” were drawn from such data points, capturing the specific actions and reflections of nurses in navigating these challenges.

### Examples of initial codes identified

- **“Cultural reluctance to express pain”**: Instances where nurses noted that patients, influenced by cultural beliefs, were reluctant to admit to pain.
- **“Family mediation in care decisions”**: Observations where family members played a central role in negotiating treatment options, including pain management.
- **“Institutional resource limitations”**: Nurses’ comments about the lack of resources, such as pain assessment tools, that hindered effective pain management.

### Clustering codes into themes

As the analysis progressed, codes were grouped into themes that illustrated broader patterns in the data:

- **“Cultural Barriers to Pain Expression”**: Codes like “cultural reluctance to express pain” and “endurance as a virtue” were clustered under this theme, emphasizing how cultural norms influence patients’ willingness to report pain.
- **“Family Involvement as a Facilitator”**: Codes such as “family mediation in care decisions” and “family advocacy for patient care” were combined to form this theme, highlighting the role of family members in supporting and advocating for pain management.
- **“Systemic Barriers to Pain Management”**: Codes like “resource limitations” and “time constraints” were grouped under this theme, focusing on the structural and institutional challenges that nurses faced in providing adequate pain management.

### Analytical reliability

To enhance the reliability of the analysis, a second experienced qualitative researcher independently reviewed a subset of the data. This researcher applied the initial coding framework to ensure consistency and objectivity in how codes were generated and applied. Discrepancies that arose during this process were discussed in collaborative meetings between the primary and second authors. These discussions aimed to reach consensus on the interpretation of the data and the coding framework. As a result, adjustments were made to the framework where necessary, ensuring that the final coding and thematic structure were grounded in the data.

The collaborative effort between the primary and second researchers not only ensured consistency in the analysis but also enhanced the credibility of the findings. The iterative nature of the analysis, combined with ongoing discussions and validation through peer review, contributed to a robust and comprehensive thematic structure that accurately represented the experiences of the participants.

### Reporting the findings

The final step in the analysis involved reporting the findings in a clear and engaging manner. Each theme was described in detail, with direct quotes from participants used to illustrate the themes and sub-themes. These quotes helped convey the depth and richness of the data, providing a nuanced understanding of the barriers and facilitators to effective pain management in elderly Arab patients. The reporting focused on highlighting the cultural, institutional, and individual factors that shaped nurses’ experiences, providing valuable insights into how pain management practices can be improved.

### Ethical considerations

The study received ethical approval from the Institutional Review Board (IRB) of the Faculty of Nursing, Tanta University, Egypt (IRB No. 475-5-2024). All participants were provided with detailed information about the study’s purpose, procedures, and their rights, including the right to withdraw at any time without penalty. Informed consent was obtained before participation, and confidentiality was assured by assigning unique identifiers to each participant. All data were securely stored, and access was restricted to the research team to ensure participants’ privacy.

### Participants’ characteristics

The characteristics of the participants (Table 1) illustrate a diverse sample of nurses, with ages ranging from 28 to 58 years. This wide age range offers a variety of perspectives, from relatively newer nurses to highly experienced professionals, which is likely to influence their approaches to pain management in elderly patients. The gender distribution of participants was predominantly female, which reflects the broader demographics of the nursing workforce in Egypt and similar healthcare systems. However, the inclusion of male nurses contributes to a more comprehensive view of the nursing care dynamics and possible differences in caregiving approaches between genders.

**Table 1:** Characteristics of the participants

Participant	Age (Years)	Gender	Education Level	Years of Nursing Experience in Outpatient Settings
N1	34	Female	Bachelor’s Degree	10
N2	46	Female	Master’s Degree	15
N3	39	Male	Bachelor’s Degree	8
N4	58	Female	Advanced Diploma	25
N5	30	Female	Bachelor’s Degree	5
N6	58	Female	Diploma	25
N7	44	Male	Bachelor’s Degree	12
N8	49	Female	Diploma	18
N9	37	Male	Bachelor’s Degree	9
N10	41	Female	Bachelor’s Degree	16
N11	28	Female	Bachelor’s Degree	7
N12	53	Female	Diploma	22

The educational backgrounds of the participants vary, with some holding diplomas, while others have earned bachelor’s or master’s degrees in nursing. This diversity in academic preparation enriches the study, as it provides insights into how different educational levels might affect approaches to pain management and patient-centered care. Nurses with higher academic qualifications, such as bachelor’s and master’s degrees, may bring more advanced theoretical knowledge, whereas those with diplomas often rely heavily on practical experience.

The participants’ years of experience in outpatient settings ranged from 5 to 25 years. This variation in experience levels allowed for the inclusion of both seasoned professionals with extensive practical knowledge and relatively younger nurses who may bring fresh perspectives to pain management practices. Such a mix of experiences is essential for a nuanced exploration of the barriers and facilitators to effective pain management in elderly patients within this cultural and clinical context.

### Thematic analysis of barriers and facilitators to effective pain management in elderly Arab patients

The following section presents a detailed account of the themes and subthemes identified from the qualitative analysis ( Table 2 ). The integration of direct participant quotes, observational data, and document analysis provides a comprehensive understanding of the barriers and facilitators experienced by nurses in managing pain among elderly Arab patients. The themes are categorized into cultural, institutional, and professional factors that shape pain management practices, highlighting the complexities and nuances of nursing in this context.

**Table 2:** Thematic results – barriers and facilitators to effective pain management in elderly Arab patients

Theme	Subtheme	Description
Cultural Barriers to Pain Expression	Stoicism and Religious Beliefs	Nurses reported that many elderly Arab patients viewed pain as a test of faith or a part of life that must be endured, leading to underreporting of pain and reluctance to seek relief.
	Family Influence	Family members sometimes discourage patients from accepting pain medications due to cultural or personal beliefs, complicating the pain management process for nurses.
Institutional Barriers to Pain Management	Resource Limitations	Nurses identified a lack of proper tools and limited staff resources as major barriers to adequately assessing and managing pain in elderly patients.
	Time Constraints	High patient loads and limited time per patient hinder nurses’ ability to provide thorough pain assessments and personalized care for effective pain management.
Facilitators to Effective Pain Management	Family Support as a Facilitator	Family members who advocated for their loved ones’ comfort facilitated more effective pain management, assisting in communication between the patient and nurse.
	Nurse Adaptability	Nurses adapted their pain management strategies based on patient preferences, employing non-pharmacological interventions when medications were refused or culturally resisted.
Interdisciplinary Collaboration	Teamwork as a Facilitator	Collaboration between nurses and other healthcare professionals such as physical therapists, pharmacists, and social workers enhanced the quality and effectiveness of pain management.
	Education and Training	Advanced pain management training allowed nurses to implement better assessment techniques and advocate more confidently for appropriate interventions.
Emotional and Professional Rewards	Emotional Satisfaction	Nurses expressed deep emotional fulfillment from successfully managing patients’ pain, highlighting patient gratitude as a key source of personal reward.
	Professional Growth	Nurses viewed the challenges of managing pain in elderly patients as opportunities for professional development, improving their skills and strategies through experience.

### Cultural barriers to pain expression

1. **Stoicism and Religious Beliefs** Cultural norms of stoicism and religious beliefs strongly influenced how elderly Arab patients expressed pain, often resulting in underreporting or refusal of pain relief. Participant N4 highlighted this, saying, “Many elderly patients believe that pain is part of life or a test from God. They think it’s something they should endure quietly, so they don’t complain as much as they should.” Similarly, Participant N2 remarked, “Some patients see their suffering as a way to earn God’s favor, so they refuse medication even when it’s obvious they are in pain.” Observational data supported these accounts, as during pain assessments, several patients demonstrated physical signs of discomfort but verbally downplayed their pain levels.
  Participant N5 also noted, “It’s hard for us as nurses to really know how much pain they’re in because they feel that showing pain is a weakness or shameful.” This sentiment was echoed during a patient-nurse interaction observed in the outpatient clinic, where a patient with chronic arthritis continuously refused stronger pain relief, stating, “It’s God’s will, I can bear it.” In patient files, nurses recorded difficulties in obtaining accurate pain scores, often noting patient hesitance or resistance to admitting the extent of their pain.

2. **Family Influence** While family members often play a supportive role in patient care, they can also act as barriers in pain management by imposing their beliefs on treatment options. Participant N7 shared, “Sometimes the family is more resistant than the patient. They don’t want their loved one to be on painkillers because they fear addiction or side effects, which complicates our job.” This was evident during observations, where family members frequently intervened during consultations, advising against certain medications or pushing for non-medical alternatives.
  Participant N9 added, “The family’s influence is huge. If they say no to a treatment, it’s hard for the patient to go against that, even if they’re in pain.” This was observed when a daughter insisted her elderly mother should not take opioids for her severe back pain, fearing dependency, despite the nurse’s recommendation for better pain management. Document analysis also showed multiple instances where family preferences were noted as a decisive factor in altering pain management plans, leading to delays or adjustments in treatment.

### Institutional barriers to pain management

1. **Resource Limitations** Nurses consistently identified a lack of adequate resources as a major institutional barrier to effective pain management. Participant N6 expressed her frustration, “We often don’t have enough tools to properly assess pain in elderly patients. Sometimes, the pain scales we use are too generic and don’t capture what they’re really feeling.” This lack of appropriate tools was evident during observations, where nurses had to rely on simple verbal pain scales that often failed to reflect the complexity of chronic pain in elderly patients.
  Participant N10 added, “In the ideal world, we would have more advanced tools, or even more time to sit down and assess pain thoroughly. But with so many patients and so few resources, we do what we can with what we have.” This resource scarcity was particularly evident in the high patient load observed during clinic hours, where nurses were pressed for time and often lacked the tools to provide more personalized pain assessments. Documentation of patient care also revealed inconsistencies in pain assessments, with nurses noting “incomplete pain evaluations” due to time and resource constraints.

2. **Time Constraints** Time limitations were another critical institutional barrier reported by participants. Participant N2 shared, “We’re expected to see so many patients in such a short time that it’s hard to give each one the attention they need, especially when it comes to something as complex as pain management.” During observations, nurses were seen juggling multiple patients at once, often hurrying through pain assessments to meet the demands of a busy outpatient schedule.
  Participant N8 echoed this sentiment, saying, “It’s a race against the clock most days. We want to sit down with each patient and really dig into what they’re experiencing, but with so many waiting outside, it’s impossible.” This was supported by document analysis, where nurse reports often cited “lack of time” as a reason for inadequate pain evaluations. The lack of time also hindered nurses from explaining treatment options thoroughly, particularly when patients were resistant to medications due to cultural beliefs.

### Facilitators to effective pain management

1. **Family Support as a Facilitator** While family influence can be a barrier, it also serves as a significant facilitator when family members actively support pain management efforts. Participant N3 noted, “When the family is on board, everything goes smoother. They help the patient understand that it’s okay to take medication for pain relief.” Observational data confirmed this, with family members sometimes stepping in to clarify the patient’s pain to the nurse, particularly when the patient was reluctant to speak about it.
  Participant N11 added, “Families can be great advocates. They often push for better pain management when they see their loved one suffering, and that really helps us.” This was reflected in several patient files, where family involvement was noted as a key factor in improving adherence to pain management plans. In one instance, a family member’s insistence on reevaluating a patient’s medication led to a more aggressive pain management plan, which significantly improved the patient’s comfort level.

2. **Nurse Adaptability** Nurses’ ability to adapt their care strategies within the constraints they face was another facilitator to effective pain management. Participant N5 shared, “We’ve learned to get creative. When a patient refuses painkillers, we might try non-drug methods like massage or guided relaxation to help them feel more comfortable.” During observations, nurses were seen adjusting their pain management approaches based on patient preferences, such as using warm compresses or repositioning patients when medications were not an option.
  Participant N9 described the importance of flexibility, stating, “It’s about finding the right balance between what the patient is willing to accept and what will effectively manage their pain. Sometimes that means thinking outside the box.” This flexibility was noted in care reports, where nurses documented using a combination of non-pharmacological interventions and patient education to achieve better pain control in reluctant patients.

### Interdisciplinary collaboration

1. **Teamwork as a Facilitator** Interdisciplinary teamwork emerged as a vital facilitator in managing pain, with nurses frequently collaborating with other healthcare professionals to provide comprehensive care. Participant N1 emphasized, “We work closely with physical therapists, pharmacists, and even social workers to manage pain. It’s a team effort.” Observations confirmed this collaborative approach, with nurses regularly consulting colleagues from other departments to adjust care plans based on holistic assessments.
  Participant N7 elaborated, “We can’t do it alone. Having the input of other professionals allows us to address the pain from different angles—whether that’s physical therapy, medication adjustments, or even mental health support.” Document analysis showed that in cases where interdisciplinary collaboration was involved, pain management outcomes were generally more positive, with better adherence to treatment plans and fewer reports of unresolved pain.

2. **Education and Training** Advanced training in pain management was another facilitator, enabling nurses to implement more effective strategies. Participant N4 explained, “My additional training in pain management has helped me identify better ways to assess pain and educate patients about their options.” This enhanced knowledge was observed during interactions where nurses with advanced training were more confident in explaining complex pain management strategies to patients and their families.
  Participant N9 noted, “The more we learn, the better equipped we are to handle the challenges. Continuous education is key to improving how we manage pain.” This sentiment was supported by document review, where nurses with specialized pain management training documented more detailed pain assessments and were able to advocate for more appropriate interventions.

### Emotional and professional rewards

1. **Emotional Satisfaction** Despite the challenges, many nurses expressed deep emotional satisfaction from their work in pain management. Participant N12 remarked, “There’s nothing more rewarding than seeing a patient who was suffering finally find relief. That’s why we do this.” Observations of nurse-patient interactions frequently revealed moments where nurses appeared visibly moved by their patients’ gratitude, often sharing smiles or words of encouragement after successful pain interventions.
  Participant N10 shared, “The gratitude we receive from patients and their families, even if it’s just a thank-you or a smile, makes all the effort worth it.” In care reports, nurses frequently highlighted instances where patients showed improvement in their pain levels as the most rewarding aspect of their job.

2. **Professional Growth** Nurses also spoke about how managing pain in elderly patients contributed to their professional growth. Participant N6 explained, “Every difficult case is a learning opportunity. It forces you to think critically and adapt, which makes you a better nurse.” Observations showed that nurses often discussed challenging cases with their peers, reflecting on what they could have done differently to improve care.
  Participant N8 added, “I feel like I’m constantly learning and growing, especially when it comes to managing pain. There’s always something new to learn.” This commitment to professional growth was documented in training records, where nurses frequently enrolled in additional pain management courses to improve their skills and stay updated on best practices.

## Discussion

This qualitative study explored the barriers and facilitators to effective pain management in elderly Arab patients from the perspective of nurses. The findings reveal a complex interplay of cultural, institutional, and professional factors that influence pain management practices in this specific context. The themes identified provide valuable insights into the challenges faced by nurses and the strategies they employ to overcome these obstacles.

### Cultural barriers to pain expression

The study highlighted significant cultural barriers to pain expression among elderly Arab patients, particularly related to stoicism and religious beliefs. Nurses reported that many patients viewed pain as a test of faith or an inevitable part of aging, leading to underreporting and resistance to pain relief interventions. This finding aligns with previous noted that cultural and religious beliefs in Arab societies often influence pain perception and expression [32]. The stoic attitude towards pain observed in this study is consistent with findings from other cultural contexts, such as those reported by Lane (2018) in their study of South Asian patients [33].

Family influence emerged as both a barrier and a facilitator to effective pain management. In some cases, family members discouraged patients from accepting pain medications due to fears of addiction or side effects, echoing findings by Al-Mutair et al. (2014) on family involvement in Arab healthcare settings [34]. However, when family members were supportive of pain management efforts, they played a crucial role in advocating for the patient and facilitating communication with healthcare providers [35]. This dual role of family influence highlights the need for nurses to engage family members effectively in pain management education and decision-making processes [36].

### Institutional barriers to pain management

Resource limitations and time constraints emerged as significant institutional barriers to effective pain management. Nurses reported inadequate tools for pain assessment and high patient loads that hindered their ability to provide thorough pain evaluations. These findings are consistent identified resource constraints as a major challenge in providing quality healthcare in Arab countries [37]. The impact of time constraints on pain management aligns with global healthcare challenges, as noted by Akbar (2019) in his review of barriers to effective pain management [38].

The lack of appropriate pain assessment tools tailored to the cultural context of elderly Arab patients is a notable finding. This gap in resources may contribute to the underassessment and undertreatment of pain in this population, as suggested by Booker and Haedtke (2016) in their study on pain assessment in older adults [39]. The development and validation of culturally appropriate pain assessment tools for elderly Arab patients could significantly improve pain management practices in this context [40].

### Facilitators to effective pain management

Despite the challenges, several facilitators to effective pain management were identified. Nurse adaptability emerged as a key factor, with nurses employing creative strategies to manage pain when faced with cultural or institutional barriers [22]. This flexibility in approach is consistent with the findings of Lim et al. (2019), who emphasized the importance of nurses’ adaptability in providing culturally competent care [41].

Interdisciplinary collaboration was highlighted as a crucial facilitator, with nurses working closely with other healthcare professionals to provide comprehensive pain management [42]. This finding supports the growing body of evidence on the effectiveness of multidisciplinary approaches to pain management in elderly patients [43]. The importance of teamwork in overcoming barriers to pain management aligns with the recommendations of the International Association for the Study of Pain (IASP) for improving pain care in low and middle-income countries [44–46].

Education and training emerged as significant facilitators, with nurses reporting that advanced pain management training enhanced their ability to assess and manage pain effectively. This finding underscores the importance of ongoing professional development in pain management [47], . The positive impact of specialized training on pain management practices suggests that investing in education could be a key strategy for improving pain care in Arab healthcare settings [48, 49].

### Emotional and professional rewards

An interesting finding of this study was the emotional satisfaction and professional growth reported by nurses engaged in pain management for elderly patients [50]. Despite the challenges, nurses expressed deep fulfilment from successfully managing patients’ pain and viewed difficult cases as opportunities for learning and development [51]. This positive aspect of pain management work has been less explored in previous literature and offers a promising avenue for future research on nurse retention and job satisfaction in challenging healthcare environments [52].

### Implications for clinical practice

The findings of this study have significant implications for clinical practice in pain management for elderly Arab patients. Firstly, the identified cultural barriers to pain expression underscore the need for culturally sensitive pain assessment and management strategies. Nurses and other healthcare providers should be aware of the influence of stoicism and religious beliefs on pain reporting and develop approaches that respect these cultural norms while ensuring adequate pain relief. The study also highlights the importance of involving family members in pain management decisions, as they can act as both barriers and facilitators. Clinicians should strive to educate and engage family members effectively to enhance pain management outcomes. Furthermore, the institutional barriers identified, such as resource limitations and time constraints, emphasize the need for healthcare institutions to prioritize pain management by allocating appropriate resources and implementing efficient pain assessment protocols. The importance of interdisciplinary collaboration in pain management suggests that clinical practice should move towards more integrated, team-based approaches to care for elderly patients with pain. A noteworthy area for future investigation involves comparing pain management experiences between nurses in rural versus urban settings. Rural settings may face greater resource constraints, limiting access to specialized pain assessment tools and support staff, which can hinder comprehensive pain management [53, 54]. Additionally, cultural adherence to traditional pain endurance may be more pronounced in rural areas, further challenging nurses. Conversely, urban settings may offer better resources and access to interdisciplinary support but might struggle with higher patient volumes and time limitations. These distinctions highlight the need for tailored approaches to pain management training and resources based on geographical location [55].

### Recommendations

Based on the study findings, several recommendations can be made to improve pain management in elderly Arab patients. Firstly, healthcare institutions should invest in cultural competence training for nurses and other healthcare providers, focusing on understanding cultural attitudes towards pain and developing culturally appropriate pain assessment and management strategies. Secondly, the development and validation of pain assessment tools specifically tailored for elderly Arab patients should be prioritized to enhance accurate pain evaluation. Thirdly, healthcare policies should be implemented to promote family-centered care, recognizing the significant role of family members in pain management decisions. This could include structured family education programs and involving family members in care planning. Fourthly, healthcare institutions should foster environments that promote interdisciplinary collaboration in pain management, including regular team meetings, shared care plans, and collaborative training sessions. Finally, continuing education programs focusing on pain management should be made readily available to nurses, enabling them to stay updated on best practices and enhance their skills in this critical area of care.

For nurses, ongoing education in culturally sensitive pain assessment techniques is essential to address patients’ reluctance to express pain due to cultural norms. Training programs can include communication strategies that respect patients’ beliefs while encouraging accurate pain reporting. For healthcare institutions, investing in resources such as culturally appropriate pain assessment tools and expanding staffing to allow more time for pain assessments would significantly improve care outcomes. Policymakers should consider frameworks that support family-centered care models, recognizing the influential role of family members in pain management decisions within Arab contexts. Policies promoting interdisciplinary collaboration can further empower nurses and improve overall patient care.

### Limitations of the study

This study has several limitations that should be considered when interpreting its findings. Firstly, the study was conducted in a single healthcare setting in Egypt, which may limit the generalizability of the findings to other Arab countries or healthcare contexts. Cultural practices and healthcare systems may vary across different Arab nations, potentially influencing pain management practices differently. Secondly, the study relied on self-reported data from nurses, which may be subject to recall bias or social desirability bias. While efforts were made to triangulate data through observations and document analysis, the primary source of information was still the nurses’ perspectives. Thirdly, the study did not include the perspectives of patients or family members, which could have provided additional insights into the barriers and facilitators of pain management. The reliance on self-reported data introduces a risk of social desirability bias, where participants may provide responses, they believe are favorable. To mitigate this, interview questions were carefully phrased to be neutral and open-ended, encouraging honest reflections. Furthermore, data triangulation was employed by corroborating interview insights with observational data and document analysis to validate reported practices and minimize bias Despite these limitations, the study provides valuable insights into the complexities of pain management in elderly Arab patients and offers a foundation for future research in this area.

## Conclusion

This qualitative study provides a nuanced understanding of the barriers and facilitators to effective pain management in elderly Arab patients from a nursing perspective. The findings reveal a complex interplay of cultural, institutional, and professional factors that influence pain management practices in this specific context. Cultural barriers, particularly related to stoicism and religious beliefs, often lead to underreporting of pain, while institutional constraints such as resource limitations and time pressures hinder thorough pain assessments. However, the study also identified important facilitators, including nurse adaptability, family support, and interdisciplinary collaboration, which can enhance pain management outcomes when leveraged effectively. The emotional and professional rewards experienced by nurses engaged in pain management highlight the positive aspects of this challenging work. By addressing the identified barriers and building on the facilitators, healthcare systems can work towards more effective, culturally sensitive pain management practices for elderly Arab patients. This study underscores the need for culturally appropriate pain assessment tools, enhanced nurse education in pain management, and policies that promote family-Centered care and interdisciplinary collaboration. Future research should focus on developing and evaluating interventions that address these identified needs, ultimately improving the quality of life for elderly Arab patients experiencing pain.

## Electronic supplementary material

Below is the link to the electronic supplementary material.

Supplementary Material 1

## Data Availability

The datasets generated during and/or analyzed during the current study are available from the corresponding author on reasonable request.
