# Supplementary material for: Barriers and facilitators to effective pain management in elderly Arab patients: a nursing perspective through a qualitative study
Source: BMC Nurs. 2024 Dec 6;23:890. doi: 10.1186/s12912-024-02523-6 (PMC11624591; doi:10.1186/s12912-024-02523-6)
Supplement: Supplementary file 1 — Supplementary Material 1 [file 12912_2024_2523_MOESM1_ESM.docx]

**Table 1 : Interview Questions on Barriers and Facilitators to Effective Pain Management in Elderly Arab Patients**

| **Question Number** | **Interview Question** |
| --- | --- |
| Q1 | Can you describe a memorable experience where you managed pain in an elderly Arab patient that posed significant challenges? How did you approach it? |
| Q2 | How do cultural beliefs and family dynamics influence the way your elderly patients express pain or accept pain management treatments? |
| Q3 | What are the main barriers you face when managing pain in elderly Arab patients, and how do you overcome these barriers in your practice? |
| Q4 | Can you share an example of how family involvement has facilitated or hindered pain management for an elderly patient in your care? |
| Q5 | How does your collaboration with other healthcare professionals (e.g., doctors, physical therapists) impact your ability to manage pain in elderly patients? |
| Q6 | What strategies do you employ to adapt pain management plans for patients who are reluctant to accept medication due to cultural or personal beliefs? |
| Q7 | How do institutional factors, such as time constraints or resource limitations, affect your ability to provide effective pain management to elderly patients? |
| Q8 | Can you discuss how your experience in managing pain in elderly patients has contributed to your professional growth or personal satisfaction as a nurse? |
| Q9 | In what ways do you believe advanced training or education in pain management has helped improve care for elderly Arab patients in your setting? |
| Q10 | How do you balance respecting cultural beliefs with the need to provide effective pain management for elderly patients? |
